# Repeated Dose 28-Days Oral Toxicity Study of *Carica papaya* L. Leaf Extract in Sprague Dawley Rats

**DOI:** 10.3390/molecules17044326

**Published:** 2012-04-10

**Authors:** Adlin Afzan, Noor Rain Abdullah, Siti Zaleha Halim, Badrul Amini Rashid, Raja Hazlini Raja Semail, Noordini Abdullah, Ibrahim Jantan, Hussin Muhammad, Zakiah Ismail

**Affiliations:** 1 Herbal Medicine Research Center, Institute for Medical Research, Jalan Pahang 50588, Kuala Lumpur, Malaysia; Email: ctzaleha.h2@gmail.com (S.Z.H.); badrul@imr.gov.my (B.A.R.); jholin_ra82@yahoo.com (R.H.R.S.); dinidtmp@yahoo.com (N.A.); sien782004@yahoo.co.uk (H.M.); drzakiah@gmail.com (Z.I.); 2 Infectious Disease Research Center, Institute for Medical Research, Jalan Pahang 50588, Kuala Lumpur, Malaysia; Email: noorrain@imr.gov.my; 3 Faculty of Pharmacy, University Kebangsaan Malaysia, Jalan Raja Muda Abdul Aziz 50300, Kuala Lumpur, Malaysia; Email: ibj@medic.ukm.my

**Keywords:** *Carica papaya*, papaya leaf, chemical fingerprinting, sub-acute toxicity study, 28-days repeated dose

## Abstract

*Carica papaya* L. leaves have been used in ethnomedicine for the treatment of fevers and cancers. Despite its benefits, very few studies on their potential toxicity have been described. The aim of the present study was to characterize the chemical composition of the leaf extract from ‘Sekaki’ *C. papaya* cultivar by UPLC-TripleTOF-ESI-MS and to investigate the sub-acute oral toxicity in Sprague Dawley rats at doses of 0.01, 0.14 and 2 g/kg by examining the general behavior, clinical signs, hematological parameters, serum biochemistry and histopathology changes. A total of twelve compounds consisting of one piperidine alkaloid, two organic acids, six malic acid derivatives, and four flavonol glycosides were characterized or tentatively identified in the *C. papaya* leaf extract. In the sub-acute study, the *C. papaya* extract did not cause mortality nor were treatment-related changes in body weight, food intake, water level, and hematological parameters observed between treatment and control groups. Some biochemical parameters such as the total protein, HDL-cholesterol, AST, ALT and ALP were elevated in a non-dose dependent manner. Histopathological examination of all organs including liver did not reveal morphological alteration. Other parameters showed non-significant differences between treatment and control groups. The present results suggest that *C. papaya* leaf extract at a dose up to fourteen times the levels employed in practical use in traditional medicine in Malaysia could be considered safe as a medicinal agent.

## 1. Introduction

*Carica papaya* L. (Caricaceae) is a shrub cultivated in many tropical, sub-tropical and temperate regions including Australia, Brazil, China, Hawaii, Malaysia and India. Many genotypes of *C. papaya* were developed to adapt to different climate, diseases infestation and to obtain desirable agronomic characteristics. The ‘Sekaki’ variety, a dwarfed shrub with a large fruit, is one of the most cultivated varieties in Malaysia, after ‘Eksotika’. Although *C. papaya *are primarily cultivated for their fruit, the juice from the green leaf is consumed as a beverage to treat malarial fever [1]. On the Gold Coast of Australia, *C. papaya* leaf juice is taken for its anti-cancer properties and recent pharmacological assessment of thejuice demonstrated anti-proliferative activity on tumor cells as well as immunomodulatory effects [2]. In French Guiana, both leaf and root are prepared in combination with *Quassia amara*, *Euterpe oleracea* and *Citrus* sp for the treatment of malarial fever [3]. *C. papaya* is also exploited for the enzyme papain, widely used in pharmaceutical and food industries [4]. 

*C. papaya* contains various compounds, including piperidine alkaloids like carpaine, pseudocarpaine, dehydrocarpaine I and II [5] and phenolics such as protocatechuic acid, *p*-coumaric acid, caffeic acid, 5,7-dimethoxycoumarin, chlorogenic acid, kaempferol and quercetin in the leaf [6], proteinases such as papain, chymopapain A and B, and endopeptidase papain III and IV in the latex and other parts of the shrub [4,7], isothiocyanate in the seed [8], non-volatile organic acids in the fruits [9], and cyanogenic compounds in the leaf, stem and fruits [10].

Despite the beneficial effects of *C. papaya* leaf, knowledge about the toxicology aspect is limited to acute toxicity data [11] and has focused on the seed and fruit. Studies on the fresh unripe fruit indicated that the ingestion of the aqueous extract caused no pathological changes in the tissues and showed no adverse effect on the functions of the liver, kidney and bone marrow in Wistar Albino rats [12]. Similar findings were observed with a methanol sub-fraction of *C. papaya* seed, which showed no systematic side effects in the studied rats [13]. Since the leaves, seed and fruit of *C. papaya* have dissimilar chemical composition, the present study aimed to characterize the low molecular weight metabolites of the leaf of *C. papaya* cultivar ‘Sekaki’ by Ultra Performance Liquid Chromatography Triple Time-Of-Flight Electrospray Interface Mass Spectrometery (UPLC-TripleTOF ESI-MS) and to determine the preclinical safety following a 28-day repeated dose oral toxicity study. 

## 2. Results and Discussion

### 2.1. Phytochemical Analysis

**Figure 1:**
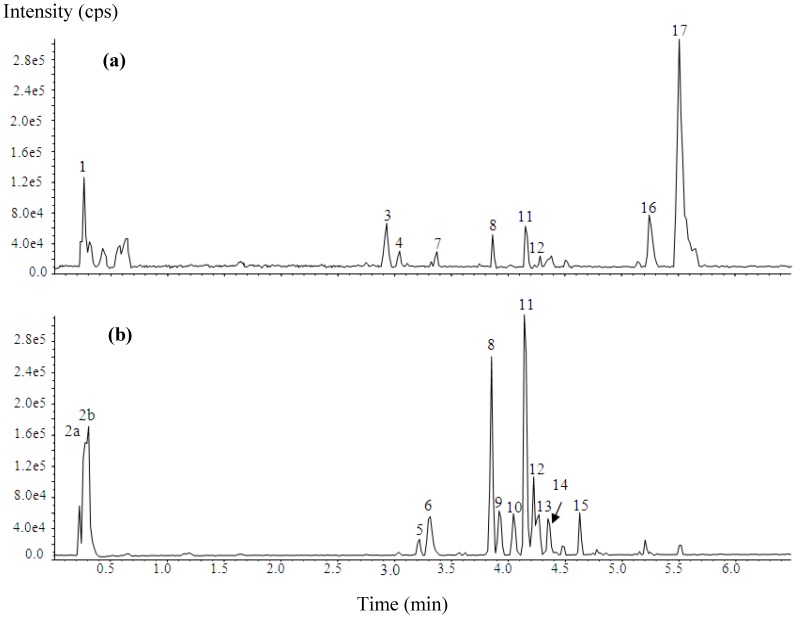
Base peak chromatograms of the studied *C. papaya* leaf extracts: (**a**) positive ionization; (**b**) negative ionization.

The UPLC-TripleTOF-ESI-MS fingerprinting of *C. papaya* leaf from cultivar ‘Sekaki’ exhibited 17 peaks, which were resolved within 6.5 min (Figure 1). Table 1 shows the retention times, pseudomolecular ion and fragmentation ions of all the compounds detected in the extract. Peak 2a exhibited a [M−H]^−^ at *m/z* 133 and the resulting MS^2^ spectrum composed of ions at *m/z* 115 [M−H−H_2_O]^−^, 89, 87, 73, 71[M−H–CO_2_–H_2_O]^−^, and 59. In keeping with the findings of Byluad [14], this peak is identified as malic acid. Peak 2b was characterized by a [M−H]^−^ at *m/z* 191, which yielded daughter ion at *m/z* 173 [M−H–H_2_O], 149, 129 [M−H–CO_2_–H_2_O], 111, 87, and 85. By reference to the mass spectrometric data of Bastos [15], peak 2b is identified as quinic acid.

**Table 1:** Compounds identified in *C. papaya* leaf extract.

No	t_R_ (min)	[M−H]^−^/[M−H]^+^ (*m/z*)	MS^2^ (*m/z*)	Assignment
1	0.26	nd/381.0846	LOW INTENSITY	Unidentified
2a	0.29	133.016/nd	133, 115, 89, 87, 73	Malic acid
2b	0.30	191.0214/nd	191, 173, 149, 129, 111, 87, 85	Quinic acid
3	2.93	nd/256.1935	256, 238, 220, 218, 122, 108	Unidentified
4	3.04	LOW INTENSITY	nd	Unidentified
5	3.22	447.1533/nd	LOW INTENSITY	Unidentified
6	3.32	295.0484/nd	295, 277, 195, 179, 146, 135, 133, 115, 89	Caffeoyl malate
7	3.37	LOW INTENSITY	nd	Unidentified
8	3.86	755.2078/757.2274	755, 301, 300, 271, 255, 179, 151	Quercetin-3-O-(2'',6''-di- *O*-rhamnopyranosyl)glucopyranoside (manghaslin)
9	3.94	279.0531/nd	279, 163, 133, 119, 115, 93	*p*-Coumaroyl malate (Isomer 1)
10	4.06	279.0535/nd	279, 163, 133, 119, 115, 93	*p*-Coumaroyl malate (Isomer 2)
11	4.16	739.2128/741.2327	739, 284, 285, 255, 227, 151, 133	Kaempferol-3- *O*-(2'',6''-di-*O*-rhamnopyranosyl)glucopyranoside (clitorin)
12	4.24	609.1479/611.1684	609, 301, 300, 271, 255, 179, 151	Quercetin-3- *O*-rutinoside (rutin)
13	4.28	309.0630/nd	309, 291, 247, 197, 193, 149, 134, 133, 115	Feruloyl malate (Isomer 1)
14	4.37	309.0637/nd	309, 291, 247, 197, 193, 149, 134, 133, 115	Feruloyl malate (Isomer 2)
15	4.64	593.1533/nd	593, 284, 285, 255, 227, 151, 133	Kaempferol-3- *O*-rutinoside (nicotiflorin)
16	5.25	nd/386.2125 ^a^	LOW INTENSITY	Unidentified
17	5.51	nd/479.3898	479, 240, 222	Carpaine

All MS^2^ were in negative ion mode except for peak no 3, 16 and 17; ^a^ [M+H]^2+^; nd: not detected.

Peaks 6, 9, 10, 13 and 14 were identified as malic acid derivatives based on the following data: (i) fragment ion at *m/z* 133, 115, 71; (ii) loss of 116 amu [M−H−133]^−^ and (iii) data reported by Harbaum [16]. Peak 6 gave [M−H]^−^ at *m/z* 295. The MS^2^ fragment ions at *m/z* 179 and 135, which based on Gomez-Romero [17] corresponds to the fragmentation of caffeic acid and thus peak 6 is tentatively identified as caffeoyl malate. Peak 9 and 10 were isomers, as both had [M−H]^−^ at *m/z* 279 which produced fragment ions at *m/z* 163 and 119, indicative of *p*-coumaroyl acid fragmentation, and thus peak 9 and 10 are tentatively identified as *p*-coumaroyl malate [17]. Other isomers, peak 13 and 14 were characterized by a [M−H]^−^ at *m/z* 309, which produced daughter ions at 193 and 149. These fragments based on the finding of Harbaum [18], is attributed to ferulic acid, and thus peaks 13 and 14 is tentatively identified as feruoyl malate isomers. 

Peaks 8 and 12 were identified as quercetin derivatives based on a significant fragment masses of the aglycone (A) at *m/z* 301 [A−H]^−^, 300 [A−2H]^−^, 179 and 151 [18,19,20]. Peak 8 had a [M−H]^−^ at *m/z* 755 and [M+H]^+^ at *m/z* 757, and the loss of 454 amu [M−H-301] suggesting two rhamnose unit (2 × 146 amu) and one glucose unit (162 amu). This was further confirmed from the NMR results as described in Section 3.2.4. The NMR data of peak 8 was consistent with data reported by Kazuma [21], and therefore is identified as quercetin-3-*O*-(2'',6''-di-*O*-rhamnopyranosyl) glucopyranoside (also known as manghaslin). Peak 12 was quercetin-3-*O*-rutinoside (also known as rutin) as it co-eluted with a standard, and both had a [M−H]^−^ at *m/z* 609 and [M+H]^+^ at *m/z* 611. 

Peak 11 and peak 15 both had the properties of kaempferol aglycone with a fragmentation peaks at *m/z* 285[A−H]^−^ and 284 [A−2H]^−^ [19]. Peak 11 exhibited [M−H]^−^ at *m/z* 739 and [M+H]^+^ at *m/z* 741. Based on the similar neutral loss of 454 amu [M−H-285]^−^ as peak 8 and supported by the NMR data reported in Section 3.2.5 as well as findings of Kazuma [21], peak 11 is identified as kaempferol-3-*O*-(2'',6''-di-*O*-rhamnopyranosyl) glucopyranoside (also known as clitorin). Peak 15 was characterized by a [M−H]^−^ at *m/z* 593. The loss of 308 [M−H-285]^−^ was indicative of one rhamnose (146 amu) and one glucose (162 amu). Thus, peak 15, in keeping with the data of Engels [19], is tentatively identified as kaempferol-3-*O*-rutinoside (also known as nicotiflorin). 

Peak 17 exhibited [M+H]^+^ at 479 and the fragmentation ions at *m/z* 240 [half molecular + H]^+^ and 222, which based on the ESI data reported by Jiao [9], is identified of carpaine.

Overall, 12 compounds consisting of one piperidine alkaloid, two organic acids, six malic acid derivatives, and four flavonol glycosides were characterized and/or tentatively identified in the leaf extract while the remaining five peaks were not identified because of the lack of information in the fragmentation pattern. Among the identified compounds, the two organic acids identified as malic acid and quinic acid as well the flavonol glycoside known as rutin and the alkaloid carpaine have been previously reported in leaf and fruit of *C. papaya* [22,23,24]. Although the phenolic acid (caffeic acid and *p*-coumaric acid) and flavonols (kaempferol and quercetin) have been previously described in the leaf extract but the identification of their ester and glycosides form was not reported [6]. In this study, caffeoyl malate, *p*-coumaroyl malate isomers and feruoyl malate isomers were identified, all of which were phenolic acids linked to malic acid by ester linkage. Both quercetin and kaempferol were not present in the aglycone form but rather as a glycosylated flavonol (rutin, manghaslin, clitorin and nicotiflorin). The manghaslin which have been regarded as the marker constituents of the highland papaya (*Carica pubescence*) cultivated in Chile was also detected in the leaf extract of cultivar ‘Sekaki’ [20]. 

The dominant compounds in the cultivar ‘Sekaki’ based on the peak area were the alkaloid carpaine and the two flavonol glycosides clitorin and manghaslin. Our findings are contrary to a previous study on the methanolic extract of African *C. papaya* leaf [6], which reported flavonols (kaempferol and quercetin) and phenolic acids (protocatechuic acid, *p*-coumaric acid and caffeic acid) as minor and major components, respectively. The presence of carpaine was not mentioned in this study but previous investigation showed that the content of alkaloid carpaine in the African variety were relatively lower than the Asian and American varieties [22]. We could not confirm whether genuine chemical variations exist in the ‘Sekaki’ as compared to the reported varieties since the differences may be due to the extraction method and choice of analytical instrument.

The glycosylated flavonols and esterified phenolic acids found in the *C. papaya* leaf extract are abundant in many dietary fruits and vegetables [25]. Evidence of the association between consumption of food containing these polyphenols with prevention of a broad range of biological properties such as cancer and cardiovascular disease is emerging and because of its naturally occurring levels in a typical human diet, its safety have been extensively studied [26,27]. There are contradictory reports concerning the safety of quercetin and kaempferol. Numerous studies have consistently reported that both compounds may be genotoxic and mutagenic but none of these effects have been demonstrated in animal or human studies [27]. Human studies have failed to demonstrate any adverse effects following oral administration of a single dose of quercetin aglycone at estimated dietary levels [26,28]. Nonetheless, as happens with most phytomedicine products, the *C. papaya* leaf extract is a complex mixture which consists of not only the glycosylated flavonols and esterified phenolics but organic acids, carpaine alkaloids and other unknown compounds and hence, in order to understand the mechanism of toxicity, a detail investigation on the pharmacokinetics and bioavailability is required. 

### 2.2. Sub Acute Study

#### 2.2.1. Clinical Observations

Following oral administration of *C. papaya* extract to male and female rats at 0.01, 0.14 and 2 g/kg BW for 28 days, no mortality and extract-related effects on the behavior and physical changes were observed. 

**Figure 2:**
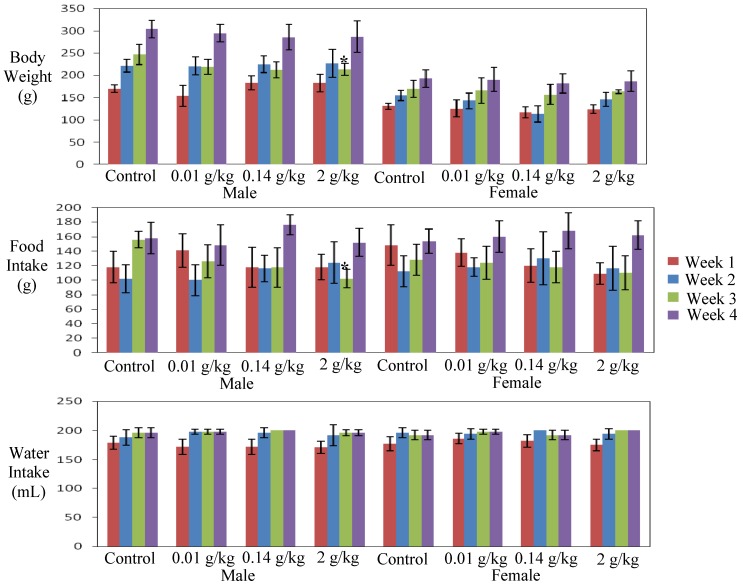
Effect of sub acute administration of *C. papaya* extract for 28 days on body weight,food intake and water intake of male and female rats. Data are expressed as mean ± standard deviation. * Significant value (*p* < 0.05).

As expected, the rat’s body weight increased with time. There was no significant difference in body weight changes between the control and treated groups, with the exception of the slightly but significantly lower body weight of the male rats treated with the highest dose (2 g/kg) at week three (*p* = 0.049). The slight decrease was not dose related, but corresponded to the significant decrease in food consumption (*p* = 0.002) (Figure 2). Food consumption and water intake in all other treated groups were not significantly different from the controls.

#### 2.2.2. Hematology, Clinical Biochemistry, Organs Weight and Histopathology Analysis

Analysis of blood parameters in animal studies is relevant to evaluate the risk of alterations of the hematological system in human toxicity [29]. The effect of sub-acute administration of *C. papaya* leaf extract on hematological parameters is presented in Table 2. Only the MCV in the male rats treated with medium dose (0.14 g/kg BW) was slightly but significantly lower (*p* = 0.039) than the controls. However, the effect was not dose dependent. Other hematological values were similar to published reference recorded for Sprague Dawley rats of 10 to 11 weeks old [30]. In contrast to previous findings, no evidence of dehydration was observed in both male and female rats as the HGB, HCT and RBC were within the normal range [11,30].

**Table 2:** Hematological findings from male and female study group, the control and rats treated with *C. papaya* leaf extracts measured during the 28 days sub-acute toxicity study. The hematological data was measured by Haematology Analyser, SYSMEX.

Sex	Parameter measured	Study group						
		Control		0.01 g/kg BW		0.14 g/kg BW		2 g/kg BW	
**Male**	WBC (10³/μL)	3.42 ± 3.60		7.14 ± 2.34		3.80 ± 4.80		7.40 ± 1.74
	RBC (10^6^×μL)	6.46 ± 0.34		6.97 ± 0.31		7.13 ± 0.41		6.97 ± 0.36
	HGB (g/dL)	13.70 ± 0.59		14.62 ± 0.56		14.38 ± 0.79		14.50 ± 0.54
	HCT (%)	41.22 ± 2.34		43.62 ± 1.88		43.38 ± 1.82		44.00 ± 1.90
	MCV (fL)	63.82 ± 0.92		62.56 ± 1.00		60.93 ± 1.05 *		63.20 ± 2.24
	MCH (pg)	21.2 ± 0.40		20.98 ± 0.40		20.18 ± 0.13		20.84 ± 0.80
	MCHC (g/dL)	33.26 ± 0.70		33.52 ± 0.54		33.13 ± 0.48		32.94 ± 0.36
	Lymphocyte %	84.70 ± 6.60		87.42 ± 1.99		82.05 ± 3.93		83.54 ± 6.65
	Lymno	3.72 ± 1.57		6.22 ± 2.00		4.25 ± 2.49		6.18 ± 1.56
	PLT(10³/μL)	1012.20 ± 220.64		1033.20 ± 139.96		997.25 ± 237.45		1023.00 ± 129.89
**Female**	WBC (10³/μL)	6.786 ± 2.021		5.880 ± 0.981		4.26 ± 4.297		6.70 ± 1.806
	RBC (10^6^×μL)	6.592 ± 0.515		6.266 ± 0.285		6.32 ± 0.164		6.612 ± 0.461
	HGB (g/dL)	13.92 ± 0.729		13.60 ± 0.32		13.578 ± 0.519		13.86 ± 0.83
	HCT (%)	41.00 ± 2.952		39.46 ± 0.976		39.36 ± 1.226		40.16 ± 2.772
	MCV (fL)	62.28 ± 2.411		63.04 ± 2.00		62.28 ± 1.04		60.78 ± 1.73
	MCH (pg)	21.16 ± 0.100		21.72 ± 0.701		21.46 ± 0.27		20.98 ± 0.593
	MCHC (g/dL)	34.00 ± 0.82		34.46 ± 0.19		34.46 ± 0.71		34.54 ± 0.38
	Lymphocyte %	81.60 ± 10.13		85.60 ± 1.685		82.78 ± 5.296		81.00 ± 6.841
	Lymno	5.40 ± 0.99		5.02 ± 0.85		4.26 ± 1.80		5.48 ± 1.75
	PLT (10³/μL)	1029.20 ± 435.12		1076.0 ± 101.53		1152.2 ± 301.92		1073.40 ± 283.31

Values are expressed as mean ± standard deviation. WBC: White Blood cells; RBC: Red Blood Cells; HGB: Hemoglobin; HCT: Hematocrit; MCV: mean corpuscular volume; MCH: mean cell hemoglobin; MCHC: mean corpuscular hemoglobin concentration; Lymno: lymphocyte number. * *p* value less than 0.05, (*p* < 0.05) significant value.

**Table 3:** Clinical biochemistry findings from male and female study group, the control and rats treated with *C. papaya* leaf extracts measured during the 28 Days sub-acute toxicity study. The Clinical biochemistry data was measured by Vitalab Selecta, E-series, Netherlands.

Sex	Parameters measured		Study Groups			
			Control	0.01 g/kg BW	0.14 g/kg BW	2 g/kg BW
**Male**		Glucose (mmol/L)	6.25 ± 1.60	10.09 ± 4.93	11.56 ± 9.51	8.13 ± 4.99
	**Liver profile**	Total Protein (g/L)	44.00 ± 7.25	48.60 ± 5.32	52.00 ± 1.41	46.20 ± 3.27
		Albumin g/L	28.74 ± 4.55	31.58 ± 2.97	32.78 ± 1.75	30.94 ± 1.99
		ALP (U/L)	253.20 ± 65.12	368.80 ± 116.41	421.75 ± 108.00 *	331.80 ± 32.67
		AST (U/L)	198.80 ± 41.44	226.00 ± 20.15	249.25 ± 75.98	232.60 ± 25.85
		ALT (U/L)	57.80 ± 11.71	81.40 ± 18.02 *	84.50 ± 5.26 *	65.80 ± 6.38
	**Renal profile**	Urea (mmol/L)	6.11 ± 1.13	6.44 ± 1.61	7.34 ± 0.80	6.56 ± 0.62
		Creatinine (μmol/L)	49.40 ± 9.69	53.80 ± 8.32	56.50 ± 16.46	51.20 ± 5.54
	**Cardiac profile**	CK (U/L)	1017.60 ± 190.33	1002.20 ± 241.34	1187.75 ± 674.08	739.40 ± 194.35
		LDH (U/L)	2555.80 ± 339.72	2526.80 ± 384.92	2069.25 ± 559.93	2327.80 ± 304.06
	**Lipid profile**	HDL-Cholesterol(mmol/L)	17.19 ± 3.79	15.39 ± 1.85	14.54 ± 1.48	14.47 ± 5.48
		Cholestrol (mmol/L)	1.29 ± 0.23	1.33 ± 0.14	1.53 ± 0.11	1.43 ± 0.29
		Triglycerides (mmol/L)	0.53 ± 0.11	0.72 ± 0.22	0.93 ± 0.15 *	0.85 ± 0.07 *
**Female**		Glucose (mmol/L)	11.12 ± 4.46	12.43 ± 4.54	13.00 ± 5.00	11.47 ± 5.15
	**Liver profile**	Total Protein (g/L)	63.20 ± 5.40	63.80 ± 2.49	78.00 ± 6.08 *	52.50 ± 9.00
		Albumin g/L	49.54 ± 15.62	42.20 ± 1.72	48.28 ± 3.19	34.76 ± 5.99
		ALP (U/L)	236.80 ± 70.05	257.20 ± 64.66	303.80 ± 119.89	225.00 ± 83.47
		AST (U/L)	43.20 ± 32.74	38.40 ± 41.37	312.80 ± 73.75 *	263.60 ± 116.75 *
		ALT (U/L)	52.52 ± 21.60	60.40 ± 4.67	88.00 ± 8.00	72.4 ± 41.42
	**Renal profile**	Urea (mmol/L)	7.39 ± 0.51	8.09 ± 1.38	8.29 ± 1.53	6.38 ± 1.24
		Creatinine (μmol/L)	63.60 ± 5.17	71.00 ± 10.49	64.20 ± 4.32	49.00 ± 6.48
	**Cardiac profile**	CK (U/L)	1220.20 ± 525.63	1207.60 ± 422.03	1505.40 ± 723.17	1220.50 ± 566.09
		LDH (U/L)	1756.00 ± 828.12	1699.20 ± 841.18	2174.00 ± 805.54	2848.75 ± 189.43
	**Lipid profile**	HDL-Cholesterol(mmol/L)	19.74 ± 3.41	22.98 ± 3.52	31.08 ± 1.71 *	19.17 ± 4.32
		Cholestrol (mmol/L)	1.68 ± 0.25	1.78 ± 0.32	2.07 ± 0.06	1.34 ± 0.25
		Triglycerides (mmol/L)	0.92 ± 0.26	1.01 ± 0.25	1.33 ± 0.32	1.07 ± 0.34

Values are expressed as mean ± standard deviation. ALP: Alkaline Phosphatase; AST: aspartate transminase; ALT: Alanine Aminotransferase; GGT: gamma glutamyl transferase; CK: Cretinine Kinase; LDH: Lactate dehydrogenase. * *p* value less than 0.05, (*p* < 0.05) significant value.

Variable changes in the biochemical parameter profile, particularly in the liver and lipid profile caused by sub-acute administration of *C. papaya* extract are shown in Table 3. The total bilirubin values (data not shown) of the male and female rats remained within the normal laboratory range. Elevation in liver enzyme activities (ALP, ALT and AST) could be regarded as a sign of damage to the hepatocellular membrane [31] and is often associated with elevation in bilirubin, urea reduction and albumin reduction [32]. The ALT level was significantly higher in the male rats treated with low and medium dose (*p* = 0.03 and *p* = 0.02, respectively), whereas the ALP level was significantly higher only in rats treated with medium dose (*p* = 0.04). At medium and high dose group of the male rats, the triglycerides were significantly higher (*p* = 0.005 and *p* = 0.018, respectively) as compared to the control group. Nevertheless, these changes were not dose-dependent and/or reflected by changes in other related parameters such as bilirubin, urea and albumin Significantly higher total protein and HDL-Cholesterol in the female rats treated with medium dose (*p* = 0.07 and *p* = 0.00, respectively) along with higher AST (*p* = 0.00 and *p* = 0.001, respectively) in the female rats from the medium and high dose groups as compared to the controls were also noted but again, the increase was not dose dependent. In addition, since the histopathological examination of the liver recovered from both male and female in all treated rats did not reveal any apparent alteration in the morphological cellular and structures, it is reasonable to infer that the biochemical changes in the liver enzyme could be regarded as incidental and not an indication of liver damage. The lack of evidence of toxicity is also strengthened by the fact that histopathology of other internal organs of all treated rats revealed no detectable abnormalities as compared to the control rats and the relative organ weight of each organ recorded at necropsy in the treatment groups did not show significant difference as compared to the control (Table 4). 

**Table 4:** The relative organ weight per 100 g body weight recorded at the end of the study from male and female rats treated with *C. papaya* extract measured during the 28 days sub-acute toxicity study.

Sex	Organ	Study Groups			
		Control	0.01 g/kg BW	0.14 g/kg BW	2 g/kg BW
**Male**	Lung	0.52 ± 0.14	0.48 ± 0.11	0.44 ± 0.05	0.49 ± 0.02
	Heart	0.34 ± 0.03	0.33 ± 0.03	0.34 ± 0.00	0.32 ± 0.02
	Liver	4.07 ± 0.36	3.56 ± 0.36	3.97 ± 0.28	3.78 ± 0.22
	Stomach	0.53 ± 0.06	0.51 ± 0.05	0.53 ± 0.05	0.53 ± 0.02
	Spleen	0.21 ± 0.05	0.12 ± 0.02	0.19 ± 0.03	0.22 ± 0.03
	GIT	0.56 ± 0.16	0.44 ± 0.06	0.45 ± 0.18	0.55 ± 0.09
	Kidney Left	0.38 ± 0.04	0.38 ± 0.05	0.39 ± 0.03	0.40 ± 0.05
	Kidney Right	0.38 ± 0.03	0.38 ± 0.04	0.40 ± 0.03	0.41 ± 0.04
	Testis Left	0.39 ± 0.16	0.49 ± 0.07	0.50 ± 0.04	0.52 ± 0.06
	Testis Right	0.42 ± 0.18	0.50 ± 0.07	0.50 ± 0.04	0.53 ± 0.04
	Adrenal Left	0.01 ± 0.01	0.01 ± 0.00	0.01 ± 0.00	0.01 ± 0.01
	Adrenal Right	0.01 ± 0.01	0.01 ± 0.01	0.01 ± 0.01	0.01 ± 0.00
**Female**	Lung	0.50 ± 0.04	0.49 ± 0.05	0.49 ± 0.05	0.46 ± 0.07
	Heart	0.37 ± 0.04	0.34 ± 0.01	0.35 ± 0.03	0.33 ± 0.03
	Liver	4.02 ± 0.21	3.87 ± 0.18	4.04 ± 0.24	3.85 ± 0.27
	Stomach	0.64 ± 0.09	0.54 ± 0.04	0.58 ± 0.10	0.56 ± 0.06
	Spleen	0.23 ± 0.02	0.21 ± 0.02	0.20 ± 0.02	0.22 ± 0.03
	GIT	0.67 ± 0.12	0.60 ± 0.13	0.61 ± 0.07	0.61 ± 0.09
	Kidney Left	0.37 ± 0.02	0.40 ± 0.01	0.39 ± 0.04	0.39 ± 0.03
	Kidney Right	0.39 ± 0.02	0.40 ± 0.03	0.39 ± 0.04	0.39 ± 0.03
	Ovary Left	0.02 ± 0.01	0.03 ± 0.01	0.03 ± 0.01	0.03 ± 0.02
	Ovary Right	0.02 ± 0.01	0.03 ± 0.01	0.03 ± 0.01	0.03 ± 0.01
	Adrenal Left	0.01 ± 0.00	0.01 ± 0.01	0.02 ± 0.00	0.01 ± 0.01
	Adrenal Right	0.01 ± 0.00	0.01 ± 0.01	0.01 ± 0.00	0.01 ± 0.00
	Urinary bladder	0.04 ± 0.01	0.03 ± 0.01	0.03 ± 0.01	0.03 ± 0.01

Values are expressed as mean ± standard deviation. * *p* value less than 0.05, (*p* < 0.05) significant value.

## 3. Experimental

### 3.1. Plant Material and Extraction

Fresh green leaf of *C. papaya* cultivar ‘Sekaki’ from the female tree were purchased from Malaysian Agricultural Research and Development Institute, Serdang, Selangor. A voucher specimen was deposited in the Forest Research Institute Malaysia, Kepong, Malaysia (Voucher No: 007/10). The leaves were washed under running tap water, separated from the midrib and extracted using a juicer (Panasonic, Shah Alam, Malaysia). The resulting juice (without addition of water) was lyophilized to obtain a dark green powder (2.6% w/w yield). Prior to chemical fingerprinting by UPLC-Triple TOF-ESI-MS, a sample of the green powder (5 mg) was dissolved in 20% acetonitrile (1 mL) and filtered with a PTFE 0.2 µm syringe filter. As for toxicology analysis in rats, the green powder was dissolved in distilled water at a concentration of 0.01, 0.14 and 2 g/kg body weight (BW).

### 3.2. Phytochemical Analysis

#### 3.2.1. Chemicals

HPLC grade methanol, MS grade acetonitrile and reagent grade formic acid (98%, v/v) were purchased from Merck Chemicals (Darmstadt, Germany). Trifluoroacetic acid (98%, v/v) was provided by Sigma Chemical Co. (St. Louis, MO, USA). Water was purified by a Milli-Q purification system from Millipore (Bedford, MS, USA). 

#### 3.2.2. UPLC-TripleTOF-ESI-MS Instrumentation and Conditions

UPLC-TripleTOF-ESI-MS analysis were performed on an AB SCIEX TripleTOF 5600-1 triple time-of-flight mass (TOF) spectrometer (AB SCIEX, Foster City, CA, USA) equipped with an electrospray interface and coupled with an Acquity UPLC system (Waters, MA, USA). Analyst TF 1.5 software (AB SCIEX) was used to control the instrument and for data processing and acquisition. The capillary and voltage of the ESI-MS source were maintained at 350 °C and 5.5 kV, respectively. All other parameters were as follows: nitrogen was used as ion source gas for nebulisation, curtain gas; 30 psi, collision gas; 10 psi, declustering potential; 80 V. Detection was performed in positive and negative ion modes in the *m*/*z *range 100–1,000. The TripleTOF mass spectrometer was calibrated for both positive and negative polarities and the error was less than 3 ppm. The separations were carried on a reversed columns (BEH C18, 1.7 µm, 2.1 × 50mm, Waters, Milford, MA, USA), which was maintained at 40 °C. The mobile phase consisted of the following 10 min sequence of linear gradient and isocratic solvents of solvent A (0.1% v/v of formic acid in water,) and solvent B (0.1% v/v of formic acid in acetonitrile) at a flow rate of 500 µL/min: 0–1.75 min, 5% B; 1.75–6.75 min, 5–30% B; 6.75–7.25 min, 30–95% B; 7.25–7.50 min, isocratic at 95% B; 7.50–8.0 min (washing step); and finally equilibrated under initial condition for 2 min.

#### 3.2.3. Isolation and Characterization of Major Flavonol Glycosides

In order to confirm the two major flavonol glycosides, multiple chromatographic procedures were performed. Initially, lyophilized *C. papaya* leaf extract (14.89 g) was subjected to repeated solid-phase extraction over reversed phase C18 cartridges (Phenomenex, Torrance, CA, USA, 5 g, 20 mL) using stepwise elution from water to methanol (water, 30% aqueous methanol, 35% aqueous methanol and methanol). The two major flavonol compounds; manghaslin (Peak 8) and clitorin (Peak 11) were fractionated in 30% aqueous methanol and was further purified by semi-preparative HPLC using a system comprising of Waters 600 controller, Waters 2487 detector, Rheodyne 7725i manual injector, Clarity software and a C-18 column (Phenomenex, Torrance, CA, USA, Luna 10 µm, 100A, 150 × 10.0 mm). The mobile phase consisted of water: trifluoroacetic acid (0.005% v/v; solvent A) and acetonitrile: trifluoroacetic acid (0.005% v/v; solvent B) with the following elution profile: 0–5 min, 10% B; 5–20 min, 10–30% B; 20–21 min, 30–95% B; 21–24 min 95% B; 24–26 min, 95–10% B; 26–30 min, 10% B. The flow rate was 5 mL/min and the chromatographic profiles were monitored at 355 nm. NMR spectroscopy was used to elucidate the structure of the two isolates by means of 1D and 2D NMR (^1^H, J-Mod, COSY, HSQC and HMBC). NMR spectra were recorded on a Bruker Avance DRX500 instrument (^1^H at 500.13 MHz; ^13^C at 125.77 MHz). All spectra were obtained in deuterated methanol (CD_3_OD) with chemical shifts expressed in ppm and coupling constant (*J*) in Hertz (Hz).

#### 3.2.4.Quercetin-3-O-(2'',6''-di-O-rhamnopyranosyl) glucopyranoside (manghaslin) (Peak 8)

^1^H-NMR (ppm): δ: aglycone: 6.19 (1H, br s, H-6), 6.38 (1H, br s, H-8), 6.87 (1H, *d*, *J* = 8.2, H-5'), 7.60 (1H, br s, H-2'), 7.62 (1H, *dd*, *J* = 2.0, 8.2, H-6'); 3-Glucosyl: 3.27 (*m*,H-4), 3.33 (*m*, H-5), 3.54 (*dd*, *J* = 8.9, 9.0, H-3), 3.65 (*dd*, *J* = 7*.*7, 9.0, H-2), 3.82 (*m*, H-6a), 3.38 (*m*, H-6b), 5.59 (*d*, *J* = 7.7, H-1); 2"-Rhamnosyl: 1.01 (*d*, *J* = 6.2, H-6), 3.35 (*m*, H-4), 3.79 (*m*, H-3), 4.00 (*m*, H-2), 4.08 (*dq*, *J* = 6.2, 9.5, H-5), 5.23 (*br s*, H-1); 6"-Rhamnosyl: 1.08 (*d*, *J* = 6.2, H-6), 3.24 (*t*, *J* = 9.5, H-4), 3.41 (*m*, H-5), 3.49 (*dd*, *J* = 3.3, 9.5, H-3), 3.58 (*m*, H-2), 4.51 (*br s*, H-1). ^13^C-NMR (ppm): δ: aglycone: 94.85 (C-8), 99.90 (C-6), 106.09 (C-10), 116.22 (C-5'), 117.59 (C-2'), 123.62 (C-6'), 123.72 (C-1'), 134.63 (C-3), 146.07 (C-3'), 149.69 (C-4'), 158.61 (C-9), 159.08 (C-2), 163.31 (C-5), 165.74 (C-7), 179.46 (C-4); 3-Glucosyl: 68.46 (C-6), 72.06 (C-4), 77.27 (C-5), 79.09 (C-3), 80.22 (C-2), 100.65 (C-1); 2"-Rhamnosyl: 17.66 (C-6), 70.11^b^ (C-5), 72.48 (C-3), 72.57 (C-2), 74.25 (C-4), 102.79 (C-1); 6"-Rhamnosyl: 17.95 (C-6), 69.87^b^ (C-5), 72.30 (C-2), 72.44 (C-3), 74.05 (C-4), 102.41(C-1). ^b^ Values could be interchanged.

#### 3.2.5. Kaempferol-3-O-(2'',6''-di-O-rhamnopyranosyl) glucopyranoside (clitorin) (Peak 11)

^1^H-NMR (ppm): δ: aglycone: 6.20 (1H, *d*, *J* = 2.0, H-6), 6.39 (1H, *d*, *J* = 2.0, H-8), 6.90 (2H, *d*, *J* = 8.9, H-3', H-5'), 8.02 (2H, *d*, *J* = 8.9, H-2', H-6'); 3-Glucosyl: 3.26 (*m*,H-4), 3.35 (*m*, H-5), 3.54 (*t*, *J* = 9.1, H-3), 3.61 (*m*, H-2), 3.82 (*m*, H-6a), 3.38 (*m*, H-6b), 5.60 (*d*, *J* = 7.6, H-1); 2"-Rhamnosyl: 0.99 (*d*, *J* = 6.2, H-6), 3.37 (*m*, H-4), 3.79 (*dd*, *J* = 3.4, 9.5,H-3), 4.00 (*m*, H-2), 4.06 (*dq*, *J* = 6.2, 9.5, H-5), 5.22 (*br s*, H-1); 6"-Rhamnosyl: 1.08 (*d*, *J* = 6.2, H-6), 3.23 (*m*, H-4), 3.42 (*dq*, *J* = 6.2, 9.5, H-5), 3.48 (*dd*, *J* = 3.5, 9.5, H-3), 3.58 (*m*,H-2), 4.50 (*d*, *J* = 1.2,H-1). ^13^C-NMR (ppm): δ: aglycone: 94.93 (C-8), 99.95 (C-6), 106.11 (C-10), 116.32 (C-3', C-5'), 123.33 (C-1'), 132.30 (C-6'), 123.30 (C-2'), 134.50 (C-3), 159.21 (C-2), 158.65 (C-9), 161.37 (C-4'), 163.30 (C-5), 165.76 (C-7), 179.46 (C-4); 3-Glucosyl: 68.46 (C-6), 72.12 (C-4), 77.26 (C-5), 79.09 (C-3), 80.08 (C-2), 100.63 (C-1); 2"-Rhamnosyl: 17.70 (C-6), 70.06 (C-5), 72.49 (C-3), 72.56 (C-2), 74.22 (C-4), 102.74 (C-1); 6"-Rhamnosyl: 17.97 (C-6), 69.89 (C-5), 72.27 (C-2), 72.49 (C-3), 73.99 (C-4), 102.44 (C-1). 

### 3.3. Sub Acute Study

#### 3.3.1. Test System and Husbandry

The test system used in this study was Sprague Dawley Rats (SD rats) aged between six to seven weeks, weighing about 90 to 100 g. Both male and female SD rats were obtained from the Laboratory Animal Resource Unit, Medical Resource Research Center, Institute for Medical Research, Kuala Lumpur, Malaysia. The use of the laboratory animals and its study design were approved by the Institutional Animal Care and Used Committee (IACUC) (ACUC No: ACUC /KKM 02 (1/2009). The handling of rats was carried out according to the Guidelines of Handling of Laboratory Animals by the Ministry of Health Malaysia [33]. The experimental rats were housed individually in a stainless-steel wire-mesh cage (6H × 11D × 16W cm) and kept for at least 5 days prior to the study. All rats were kept in a room and maintained at room temperature and humidity of about 27 ± 2 °C and 65.85 ± 6.76 respectively with 12 h alternate artificial and natural light and dark cycle. The room temperature and relative humidity were recorded daily using a temperature datalogger (TempRH Datalogger BG-DL-01/01B). Throughout the study, the rats were given unlimited supply of clean water and fed with pellet diet; Zeigler Rodent NIH-31 irradiated Auto wafer feeds (Zeigler Bros, Gardners, PA, USA).

#### 3.3.2. Experimental Design

The sub acute toxicity study was carried out according to the OECD Guidelines No. 407 for the “Repeated Dose 28-Day Oral Toxicity Study in Rodents” [34] with some modifications, namely the temperature and humidity of the room. The rats were assigned randomly to four groups, one control group and three treatment groups consisting of low, medium and high dose level. Ten rats, five male and five female weighing about 90 to 100 g were assigned to each group. The individual body weights were within ±20% of the mean of each sex. The rats were approximately seven weeks old at the initiation of the study (initiation of dosing). The treatment group received *C. papaya* leaf extract diluted in water to the dose required while the control group received water only. 

#### 3.3.3. Selection of Doses

The high, medium and low dose levels selected in the present study were 2, 0.14 and 0.01 g/kg BW respectively. The highest dose was determined as 2 g/kg BW since at this level *C. papaya* leaf extract did not produce any acute toxicity effect [11]. The medium (therapeutic dose) and low dose level was selected based on the local traditional preparation of *C. papaya* juice for treatment of fever in human, which recommends two tablespoon of *C. papaya* leaf juice from two leaf. From the juice extraction process in the laboratory, two pieces of medium size leaf afforded 16 mL juice, which after lyophilized yielded 10 g of dried extract. The therapeutic dose of 0.14 g/kg BW was calculated based on an average weight of a human (70 kg) taking 10 g of extracts. This dose was 14 fold lower than the highest dose. Using the same common ratio, the lowest dose was selected at 0.01 g/kg BW.

#### 3.3.4. Oral Administration of the Extracts

The test group were given the *C. papaya* extract at 0.01 g/kg BW (low dose level), 0.14 g/kg BW (medium dose level) and 2 g/kg BW (high dose level) delivered in a 2 mL volume using intubation needle given daily at about the same time for 28 days. The rats were weighed weekly and the amount of *C. papaya* leaf extract were re-calculated based on the new body weight to ensure a constant dose volume per kg BW at all times. Control rats received the same volume of drinking water as the amount given to the test groups. 

#### 3.3.5 Parameters Measured during the Study

Clinical observations were made once a day for mortality, moribund and ill health or reaction to treatment, such as changes on skin and fur, eyes and mucus membrane, behavior pattern, tremors, salivation, diarrhea, sleep and coma. Individual rats were weighed before the commencement of the experiment and then weighed once on the seventh day of every week. Final body weights were recorded prior to the scheduled necropsy. The food and water were measured weekly and the differences were calculated and regarded as food and water consumption (g/rat/week). 

At the end of Day 28, all the rats were fasted and at necropsy day, Day 29, blood was drawn by cardiac puncture or whenever possible from the posterior vena cava from all rats under ether anesthesia. Blood was collected in two types of tubes, one with anticoagulant (EDTA) and the other without any additives. The anticoagulated blood (EDTA) was analyzed immediately for haematology parameter using a SYSMEX KX-21N Hematology Analyser (Sysmex Cooperation, Kobe, Japan). The hematology values measured were haemataocrit (HCT), haemaglobin concentration (HB), erythrocyte counts (RBC), total and differential leucocyte count (WBC), mean cell volume (MCV), mean cell haemoglobin (MCH), mean corpuscular haemoglobin concentration (MCHC), Lymphocyte percentage, lymphocyte number and platelet. 

The blood without any additives was for clinical biochemistry study. It was allowed to stand for a minimum of 3 h for complete clotting. The serum was collected and transferred into another tube after centrifugation at 4,000 rpm at 4 °C for 10 min. The serum was then kept at −20 °C until analysis for clinical biochemistry measurements using the Vitalab Selecta, E-series (Netherlands). Clinical Biochemistry values (Total Protein, Albumin, Total Bilirubin, Alkaline Phosphatase (ALP), Alanine Aminotransferase (ALT), Aspartate Aminotransferase (AST), Gamma Glutamyltransferase (GGT), Urea, Creatinine, Creatinine Kinase (CK), lactate dehydrogenase (LDH), glucose, HDL-Cholesterol, Cholesterol and Triglycerides were determined. 

After the withdrawal of blood, the rats were then sacrifices with an overdose of ether. Complete gross post mortem examination were carried out. The rats were dissected and careful examination of the organs (for pathological changes) was carried out. The organs including the liver, kidneys, adrenals, testes, spleen, lungs, stomach, intestine and heart were examined, removed and weighted. The relative organ weight (ROW) of each organ was then calculated as follows:


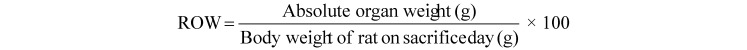


The organs were then preserved in 10% buffered formalin for subsequent histopathological examination. All organs recovered from the necropsy were preserved in 10% buffered formalin for further histopathological procedures. The tissue histopathological examination was carried out using a light Microscope Olympus BX 51 (Tokyo, Japan). 

#### 3.3.6. Statistical Analysis

Data from sub acute study were analyzed using SPSS 14.0 for windows software. A one way ANOVA and Tukey test were used to compare any significant differences between the control and therapeutic groups. Values were expressed as mean value (x), standard deviation (SD) and standard error of the mean (SEM). Differences were considered significant at *p* < 0.05.

## 4. Conclusions

UPLC-TripleTOF MS analysis revealed that the leaf extract of *C. papaya* cultivar ‘Sekaki’ contains considerable amounts of carpaine, malic acid, quinic acid, manghaslin and clitorin, minor quantities of various malic acid derivatives, nicotiflorin, rutin and unidentified unknown constituents. These complex mixtures did not produce treatment related changes in body weight, food intake, water level, hematological parameters and serum biochemistry in Sprague Dawley rats after oral sub-acute exposure for 28 days. It should be noted that although elevations (not in a dose dependent manner) in the ALT and ALP level of the male rats and increase in AST and total protein in female rats were observed, the histopathological examination of the liver showed no morphological disturbances. The relative internal organ weights including the liver showed non-significant differences between the treatment and control groups. Therefore, the current results suggest that at dose of up to 2 g/kg (14 times the dose consumed empirically in traditional medicine in Malaysia) *C. papaya* cultivar ‘Sekaki’ leaf extract are considered relatively non-toxic. Nevertheless, further chronic toxicity studies are necessary to precisely establish the oral toxicity profile.

## Supplementary Materials

Supplementary materials can be accessed at: http://www.mdpi.com/1420-3049/17/4/4326/s1.
