# Supplementary material for: Repeated Dose 28-Days Oral Toxicity Study of Carica papaya L. Leaf Extract in Sprague Dawley Rats
Source: Molecules. 2012 Apr 10;17(4):4326–42. doi: 10.3390/molecules17044326 (PMC6268730; doi:10.3390/molecules17044326)

Supplementary

Repeated Dose 28-Days Oral Toxicity Study of *Carica papaya* L. Leaf Extract in Sprague Dawley Rats

Figure 1. Histological structure of liver from female SD rat from control group showing the Central vein (CV), hepatic vein (HV), the sinusoid and the hepatocytes are normal. ×40 (H&E Staining).

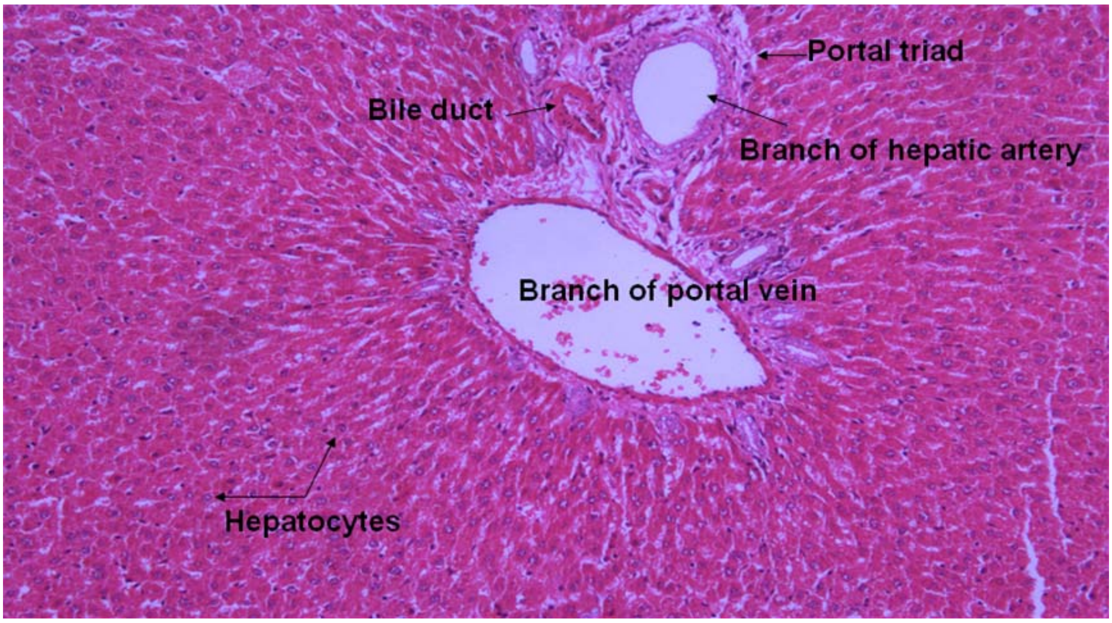

Figure 2. Histological structure of liver from female SD rat from Medium Dose group showing the Central vein (CV), hepatic vein (HV) and the sinusoid. The sinusoid and the hepatocytes were normal as compared to the control. × 20 (H&E Staining)

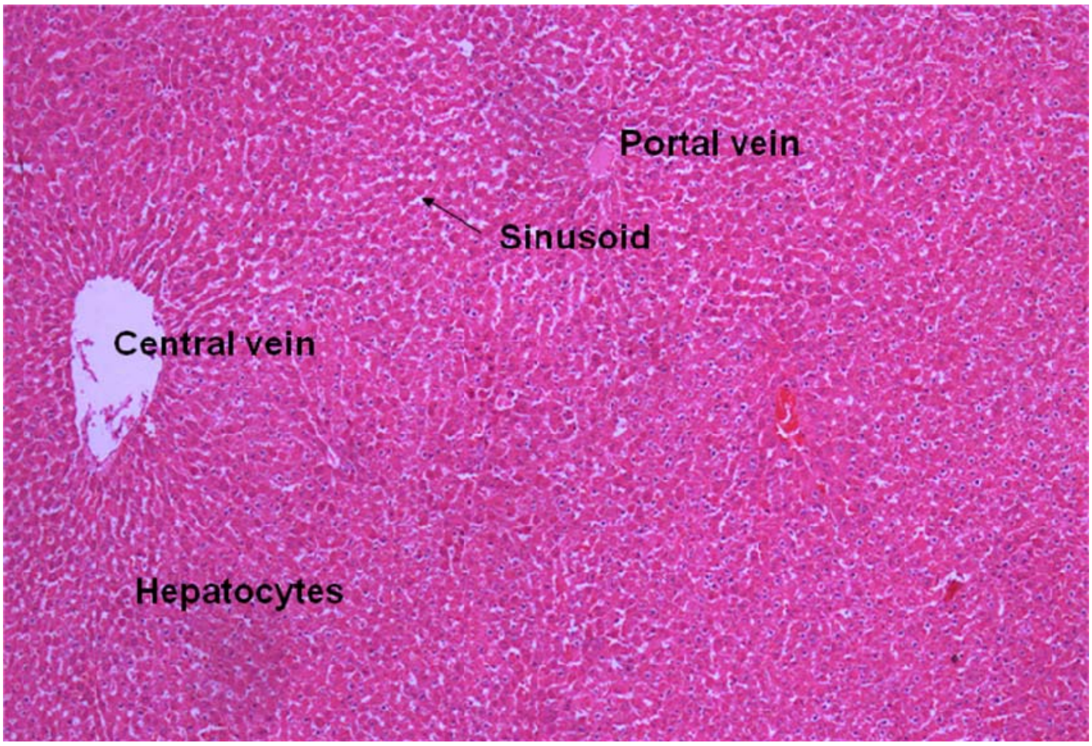

Figure 3. Histological structure of liver from female SD rat from High Dose group showing the Central vein (CV), hepatic vein (HV) and the sinusoid and the hepatocytes. The sinusoid were normal as compared to the control. (H&E Staining) ×20

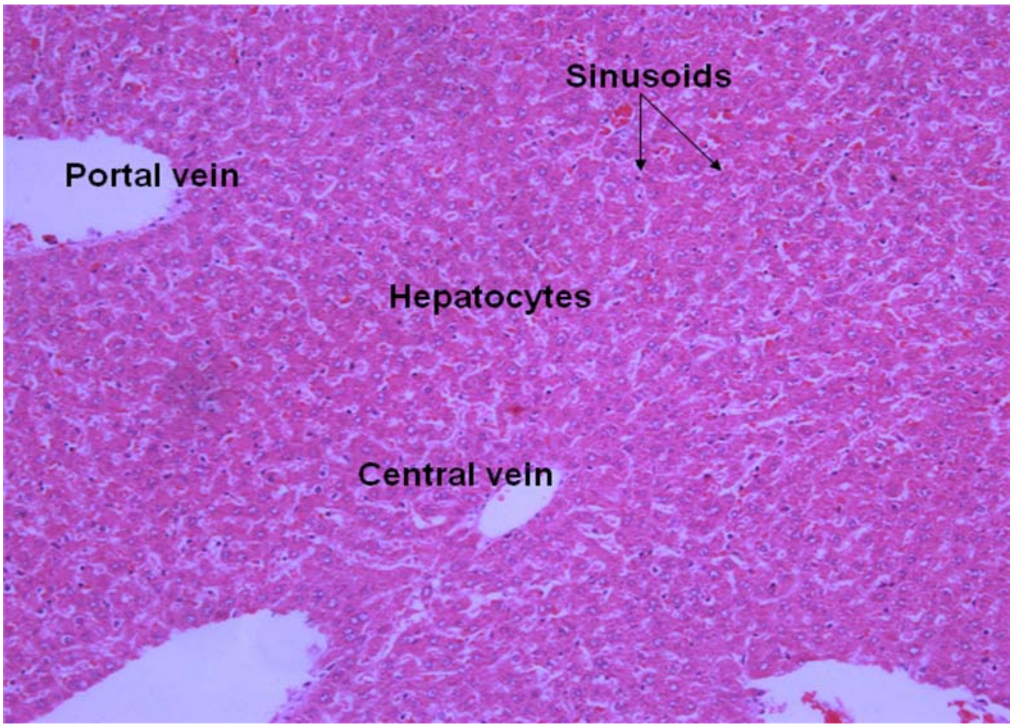

Figure 4. Histological structure of liver from female SD rat from High Dose group showing the Central vein (CV), hepatic vein (HV) and the sinusoid and the hepatocytes were normal as compared to the control. ×20(H&E Staining)

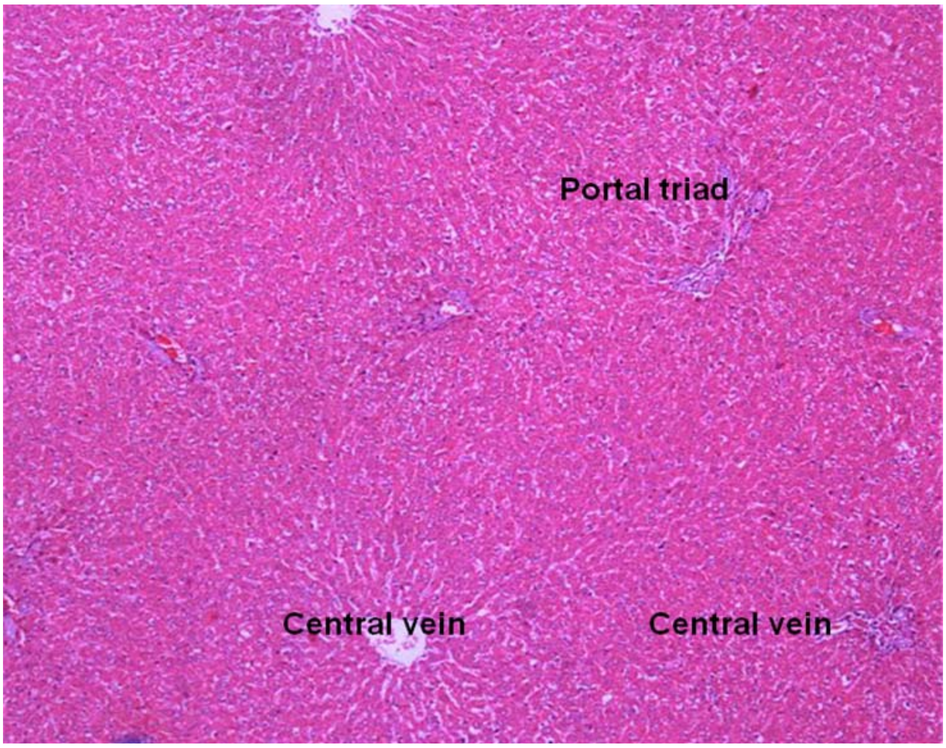

Supplement: Supplementary file 1 [file molecules-17-04326-s001.pdf]
